# Sinensetin Reduces Osteoarthritis Pathology in the Tert-Butyl Hydroperoxide-Treated Chondrocytes and the Destabilization of the Medial Meniscus Model Mice *via* the AMPK/mTOR Signaling Pathway

**DOI:** 10.3389/fphar.2021.713491

**Published:** 2021-07-16

**Authors:** Wenxian Zhou, Yifeng Shi, Hui Wang, Caiyu Yu, Huanqing Zhu, Aimin Wu

**Affiliations:** ^1^ Department of Orthopaedics, The Second Affiliated Hospital and Yuying Children’s Hospital of Wenzhou Medical University, Wenzhou, China; ^2^ Zhejiang Provincial Key Laboratory of Orthopedics, Wenzhou, China; ^3^ The Second School of Medicine, Wenzhou Medical University, Wenzhou, China; ^4^ The School of Ophthalmology and Optometry, Wenzhou Medical University, Wenzhou, China

**Keywords:** osteoarthritis, autophagy, apoptosis, AMPK/mTOR signaling pathway, sinensetin

## Abstract

As a common degenerative disease, osteoarthritis (OA) usually causes disability in the elderly and socioeconomic burden. Previous studies have shown that proper autophagy has a protective effect on OA. Sinensetin (Sin) is a methylated flavonoid derived from citrus fruits. Studies have shown that Sin is a good autophagy inducer and has shown excellent therapeutic effects in a variety of diseases; however, its role in the treatment of OA is not fully understood. This study proved the protective effect of Sin on OA through a series of *in vivo* and *in vitro* experiments. *In vitro* experiments have shown that Sin may inhibit chondrocyte apoptosis induced by tert-butyl hydroperoxide (TBHP); at the same time, it might also inhibit the production of MMP13 and promote the production of aggrecan and collagen II. Mechanism studies have shown that Sin promotes chondrocyte autophagy by activating AMPK/mTOR signaling pathway. On the contrary, inhibition of autophagy can partially abolish the protective effect of Sin on TBHP-treated chondrocytes. *In vivo* experiments show that Sin may protect against DMM-induced OA pathogenesis. These results provide evidence that Sin serves as a potential candidate for the treatment of OA.

## Introduction

Osteoarthritis (OA) is the most common degenerative disease, involving the entire joint, causing pain, and eventually leading to disability, which causes huge socioeconomic burden (Vina and Kwoh, 2018; Safiri et al., 2020). The pathological features of OA are progressive destruction of articular cartilage, chronic inflammation of the synovium, and subchondral bone sclerosis (Vina and Kwoh, 2018). Chondrocytes are the only cell type in articular cartilage, and pathological factors such as inflammation and oxidative stress lead to excessive apoptosis of chondrocytes, reduced cell density, and degradation of extracellular matrix (ECM), thereby aggravating the development of OA (van den Bosch, 2021; Goutas et al., 2018). In addition, ECM is an essential components for maintaining function and structure of cartilage (Kronenberg, 2003). Therefore, preventing excessive apoptosis of chondrocytes may be an effective treatment method to prevent the progression of OA.

Autophagy is a protective mechanism through engulfing and degrading misfolded proteins and senescent or damaged organelles to realize the metabolic needs of the cell itself and the renewal of some organelles (Klionsky et al., 2016; Mizushima, 2007). Autophagy occurs in both physiological and pathological conditions. A large number of researches showed that autophagy plays an important role in the homeostasis of articular cartilage (Caramés et al., 2015; Li et al., 2016). Autophagy enhanced by specific deletion of mTOR could significantly protect OA induced by destabilisation of medial meniscus (DMM) (Zhang et al., 2015), indicating that autophagy plays a protective role in OA.

Sinensetin (Sin) is a polymethoxylated flavonoid, which is found in citrus fruits and possess potent anti-inflammatory, anti-angiogenesis and anticancer activities (Han Jie et al., 2021). Sin is also involved in the regulation of a variety of signaling pathways, such as NF-kappaB, AKT/mTOR and MAPKs signaling (Tan et al., 2019; Li et al., 2020). A recent study showed that Sin promotes autophagy through AMPK/mTOR signaling pathway to induce hepatocellular carcinoma cell apoptosis (Kim et al., 2020). Although studies have conducted extensive studies on the function of Sin, its effect on chondrocyte autophagy and its potential therapeutic effect on the progression of OA remain unclear.

In this study, we investigated the effect of sin on chondrocyte autophagy under tert-butyl hydroperoxide (TBHP) treatment and explored its potential molecular mechanism. We also used the DMM mouse model to study the *in vivo* effects of Sin on OA.

## Materials and Methods

### Ethics Statement

All experimental procedures in this study, such as surgery, treatment, and post-operative care of animals, followed the “Guidelines for the Care and Use of Laboratory Animals” issued by the National Institutes of Health. All animal experiments were approved by the Animal Care and Use Committee of Wenzhou Medical University. No clinical trial was involved in this study.

### Reagents and Chemicals

Sinensetin (purity >99%), 3-methyladenine (3-MA), chloroquine (CQ), and bafilomycin A1 were purchased from MCE (Monmouth Junction, NJ, United States). *t*ert-butyl hydroperoxide (TBHP) solution was purchased from Sigma-Aldrich (St. Louis, MO, United States). Safranin-O, dimethyl sulfoxide (DMSO), and collagenase II were purchased from Solarbio (Beijing, China). The primary antibodies against aggrecan, collagen II, MMP13, p62, Bcl-2, Bax were obtained from Abcam (Cambridge, MA, United States); antibodies against AMPK, p-AMPK, mTOR, p-mTOR, cleaved caspase 3, LC3 antibodies against Cell Signaling Technology (Danvers, MA, United States); antibodies against Beclin1 and GADPH were acquired from Proteintech (Chicago, IL, United States). Cell counting kit-8 (CCK8) was obtained from m Dojindo (Kumamoto, Japan). Alexa Fluor 488-labeled and Alexa Fluor 594-labeled goat anti-rabbit/mouse IgG H&L secondary antibodies were acquired from Abcam (Cambridge, MA, United States). 4,6-Diamidino-2-phenylindole (DAPI) was purchased from Yeasen Biochemical (Shanghai, China). The reagents for culturing cartilage were obtained from Gibco (Grand Island, NY, United States).

### Primary Mice Chondrocytes Extraction and Culture

Under aseptic conditions, the knee joint cartilages of immature C57BL/6 mice were collected, completely cut into small pieces, washed three times with phosphate-buffered saline (PBS), and digested with 2 mg/ml collagenase II at 37°C for 4 h. Subsequently, the digested cartilage tissue was centrifuged, washed three times with PBS, and resuspended in DMEM/F12 supplemented with 10% fetal bovine serum (FBS) and 1% antibiotics (streptomycin/penicillin) in 5% CO_2_ at 37°C. Replace with fresh medium every other day. All experiments used second-passage cells to ensure consistent cell phenotypes.

### Cell Viability Assay

The CCK8 kit was used to detect the effect of Sin on the viability of chondrocytes. According to the manufacturer’s protocol, the chondrocytes of the same generation were seeded in 96-well plates (5*10^4^ cells per well) and cultured for 24 h, and then treated with different concentrations of Sin (0, 10, 20, 30, 40, and 50 μM) for 24 h. Then, the cells were washed with PBS and incubated with 100 μl of serum-free DMEM/F12 medium containing 10% CCK8 solution for 2 h at 37°C. The absorbance was measured at 450 nm using an ultraviolet spectrophotometer (Thermo Fisher). In addition, we also detected the effect of 40 μM Sin on chondrocyte viability in different time points (0, 3, 6, 12, 24, and 48 h), and the required steps are the same as above.

### Western Blotting

The chondrocytes were lysed with RIPA lysis buffer containing 1% phenylmethanesulfonyl fluoride (PMSF) for 15 min, and then centrifuged at 12,000 rpm and 4°C for 30 min to extract total cell protein. Use the BCA reagent (Beyotime, Shanghai, China) to detect protein concentration. Then, 40 ng protein was separated using 8–12% SDS-PAGE gels and transferred to polyvinylidene fluoride (PVDF) membranes (Bio-Rad, California, United States). The membranes were blocked with 5% skimmed milk for 2 h at room temperature, and washed three times with Tris-buffered saline with 0.1% Tween-20 (TBST). Then the membranes were cut, placed in the corresponding primary antibodies, and incubated overnight at 4°C. Next, the membranes were washed three times with TBST and incubated with the corresponding secondary antibody at room temperature for 90 min. Finally, the membranes were washed three times with TBST, and the membrane blots were developed using super-sensitive ECL chemiluminescence kit, and visualized by the ChemiDoc XRS + Imaging System (Bio-Rad). Quantitative analysis was performed by ImageJ software.

### Immunofluorescence

The chondrocytes were seeded in a 6-well plate for 24 h, and then treated with TBHP (30 μM) or treated with TBHP and Sin (40 μM) for 24 h. Subsequently, chondrocytes were washed three times with PBS, fixed with 4% paraformaldehyde for 15 min, and then infiltrated with 0.1% Triton X-100 in PBS for 15 min. After blocking the cells with 5% bovine serum albumin at 37°C for 1 h, they were incubated with the corresponding primary antibody [MMP13 (1:200), cleaved caspase 3 (1:100), and LC3 (1:200)] overnight at 4°C. Finally, the cells were incubated with Alexa Fluor 488 or Alexa Fluor 594 conjugated secondary antibodies for 1 h at room temperature, and stained with nuclear staining dye DAPI for 5 min. A Nikon ECLIPSE Ti microscope (Nikon, Tokyo, Japan) was used to observe the stained cells under five random different fields for each slide. Fluorescence intensity was measured with ImageJ.

### TUNEL Staining

TUNEL staining was used to detect the level of DNA damage of chondrocytes. According to the manufacturer’s protocol, the chondrocytes were fixed, stained with *in situ* cell death detection kit (Promega Corporation, Wisconsin, United States) at 37°C for 30 min, and the nuclei were stained with DAPI. Twenty-five fields of view were randomly selected from each slide, and TUNEL positive cells were counted under a Nikon ECLIPSE Ti microscope (Nikon, Tokyo, Japan).

### Animal Models

Thirty ten-week-old C57BL/6 male mice were purchased from the Shanghai Animal Center of the Chinese Academy of Sciences and randomly divided into sham, DMM and DMM + Sin groups (*n* = 10 per group). The mouse osteoarthritis model was caused by the destabilization of the medial meniscus (DMM) through surgery (Glasson et al., 2007). In brief, mice were anesthetized by intraperitoneal injection of 2% (w/v) pentobarbital (40 mg/kg), the right knee joint capsule was cut inside the patellar tendon, and the tibial ligament of the medial meniscus was cut with microsurgery scissors. Meanwhile, the same operation was performed on the left knee. The joint incision without resection of the medial meniscus ligament was used as the sham group. After that, mice in the sham group and DMM group were given saline by gavage every day, while mice in the DMM + Sin group received 50 mg/kg/day Sin dissolved in saline by gavage. Eight weeks after the operation, the mice were sacrificed, and the knee joints were collected for imaging and histological evaluation.

### X-Ray Imaging Analysis

X-ray examinations were performed on mice in all groups 8 weeks after operation. A digital X-ray machine (Kubtec Model XPERT.8; KUB Technologies Inc.) was used to perform X-ray imaging of mice at 50 Kv and 160 μA to evaluate the joint space and cartilage surface calcification changes.

### Immunohistochemical Assay

The knee joint was embedded in paraffin, sectioned, deparaffinized and hydrated, and the endogenous peroxidase was blocked by 3% hydrogen peroxide for 15 min. Then, the sections were incubated with 0.4% pepsin (Biotech, Shanghai, China) in 5 mM HCl at 37°C for 20 min for antigen retrieval. Next, the sections were incubated with 5% bovine serum albumin at room temperature for 30 min, then with the corresponding primary antibody [MMP13 (1:100), and LC3 (1:100] overnight at 4°C, and finally incubated with the HRP-conjugated secondary antibody for 1 h. The rate of positive cells was quantitated by researchers who were blinded to the experimental group. Five mice in each group were quantitatively analyzed.

### Histopathological Analysis

The three groups of mouse joint specimens were sliced and stained with Safranin O (S-O) to evaluate the destruction of articular cartilage. Another group of experienced histology researchers examined the cellularity and morphology of cartilage and subchondral bone with a microscope in a blinded manner, and used the Osteoarthritis Research Society International (OARSI) to evaluate the degree of cartilage degeneration as described previously (Glasson et al., 2010).

### Statistical Analysis

The results are presented as mean ± S.D. and are from three independent experiments. SPSS 20.0 (IBM, Armonk, NY, United States) was used for statistical analysis. Parametric data were analyzed by one-way analysis of variance (ANOVA), and then the Tukey’s test was used to compare the groups. Nonparametric data (OARSI scores) were compared by the Kruskal–Wallis H test. *p* < 0.05 was considered significant.

## Results

### Effect of Sin on Chondrocyte Viability

**FIGURE 1:**
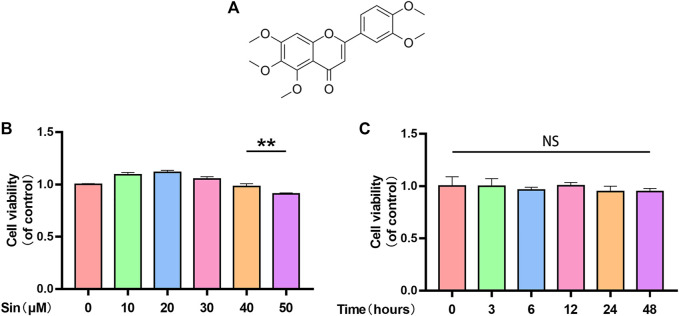
Sin’s effect on chondrocyte viability. **(A)** Chemical structure of Sin. **(B)** The results of CCK-8 assay show the viability of chondrocytes after treatment with 0, 10, 20, 30, 40, and 50 μM Sin for 24 h. **(C)** The results of CCK-8 assay show the viability of chondrocytes after treatment with 40 μM Sin for 0, 3, 6, 12, 24, and 48 h. The data are presented as the means ± SD (*n* = 3); *p < 0.05, **p < 0.01, and ***p < 0.001.

The chemical structure of Sin is shown in Figure 1A. To evaluate the toxicity of Sin to chondrocytes, we used the CCK-8 assay to determine the viability of chondrocytes treated with different concentrations of Sin (0, 10, 20, 30, 40, and 50 μM) for 24 h. The results showed that Sin concentrations below 40 μM had no obvious cytotoxicity to chondrocytes, but 50 μM Sin reduced the viability of chondrocytes (Figure 1B). Meanwhile, the cytotoxicity of 40 μM Sin at different time points (0, 3, 6, 12, 24, and 48 h) was also determined, and it was found that 40 μM Sin had no significant effect on the viability of chondrocytes within 48 h (Figure 1C).

### Effect of Sin on Apoptosis in TBHP-Treated Chondrocytes

**FIGURE 2:**
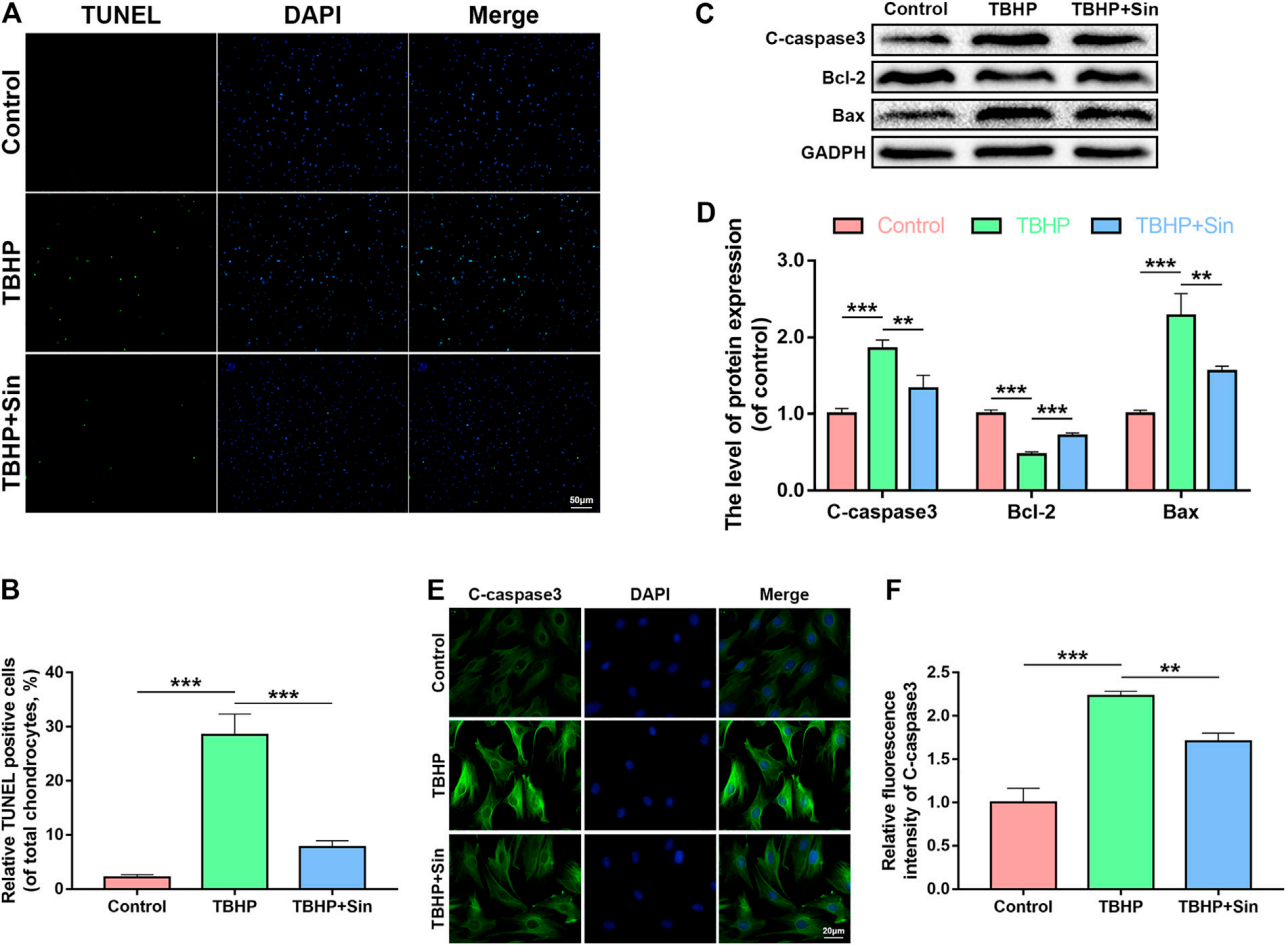
Sin protects TBHP-treated chondrocytes from apoptosis. **(A, B)** The results of TUNEL assay show the number of TUNEL-positive chondrocytes treated with or without 40 μM Sin for 24 h and 30 μM TBHP for 24 h (Scale bar: 50 μm). **(C, D)** The results of western blotting show the levels of cleaved caspase 3, Bcl-2 and Bax proteins in the chondrocytes as treated above. **(E, F)** The results of immunofluorescence show the intensity of cleaved caspase 3 in the chondrocytes as treated above (Scale bar: 20 μm). The data are presented as the means ± SD (*n* = 3); *p < 0.05, **p < 0.01, and ***p < 0.001.

TBHP significantly reduces the viability of chondrocytes (Supplementary Figure 1). We analyzed whether Sin protects chondrocytes from apoptosis through the TdT-mediated dUTP Nick-End Labeling (TUNEL) assay, immunofluorescence and western blotting analysis of the expression of apoptosis-related proteins, cleaved caspase 3 (C-caspase3), Bcl-2 and Bax. As shown in Figures 2A,B, the number of TUNEL-positive chondrocytes increased after TBHP treatment, but significantly decreased in the Sin treatment group. Meanwhile, TBHP treatment upregulated the expression of cleaved caspase 3 and Bax, and downregulated the expression of Bcl-2, while Sin treatment could reverse these conditions (Figures 2C,D). Similarly, the results of cleaved caspase 3-labeled immunofluorescence further confirmed that Sin treatment reduces the intensity of cleaved caspase 3 (Figures 2E,F). These results indicate that Sin protects chondrocytes from TBHP-induced apoptosis.

### Effect of Sin on ECM Degradation in TBHP-Treated Chondrocytes

**FIGURE 3:**
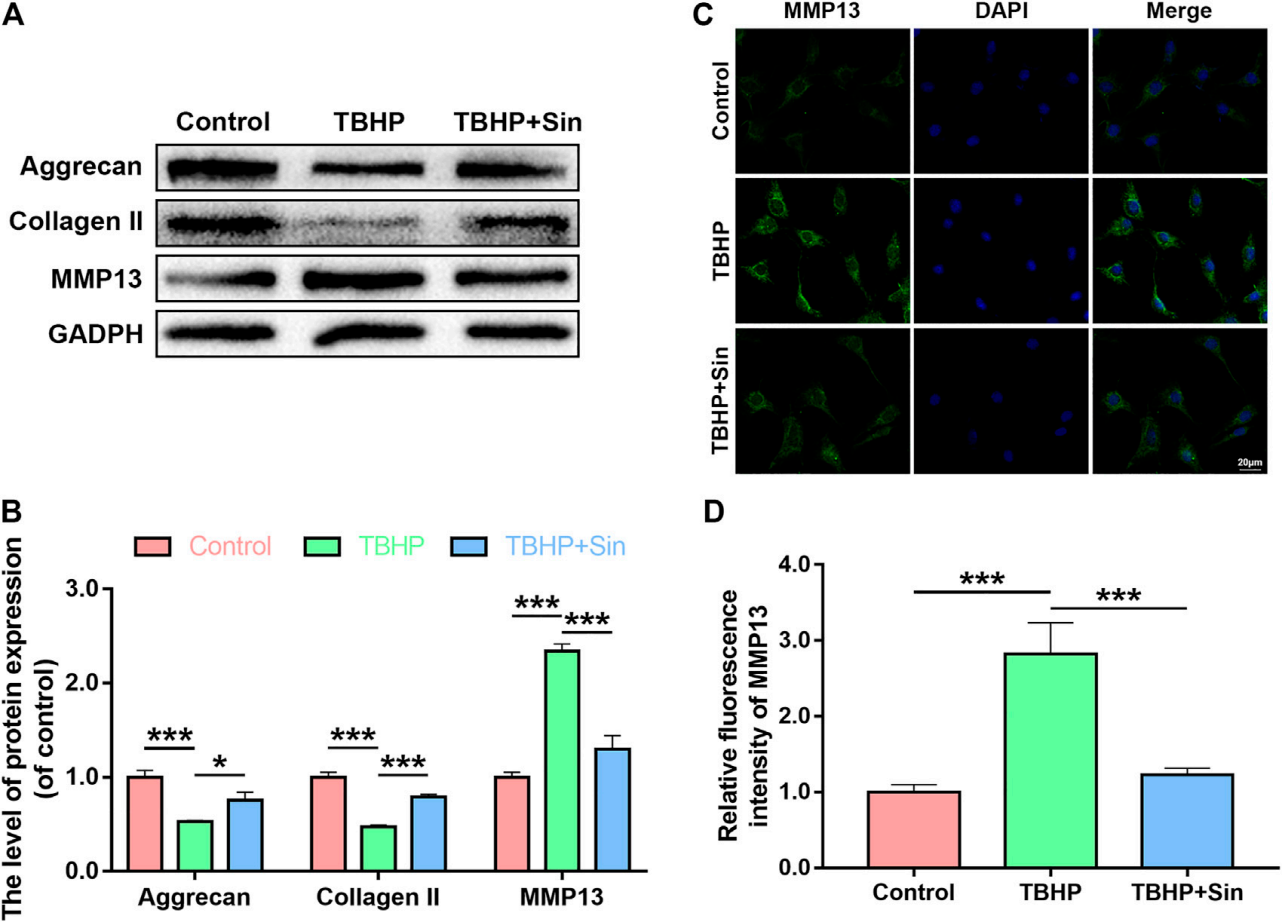
Sin protects TBHP-treated chondrocytes from ECM degradation. **(A, B)** The results of western blotting show the levels of aggrecan, collagen II and MMP13 proteins in the chondrocytes treated with or without 40 μM Sin for 24 h and 30 μM TBHP for 24 h. **(C, D)** The results of immunofluorescence show the intensity of MMP13 in the chondrocytes as treated above (Scale bar: 20 μm). The data are presented as the means ± SD (*n* = 3); *p < 0.05, **p < 0.01, and ***p < 0.001.

Next, we evaluated the effect of Sin on TBHP-induced ECM degradation by detecting the expression of collagen II, aggrecan and matrix metallopeptidase 13 (MMP13). Western blotting showed that TBHP treatment significantly reduced the expression of aggrecan and collagen II, and increased the expression of MMP13, while Sin could reverse these effects induced by TBHP (Figures 3A,B). Additionly, the results of immunofluorescence labeled with MMP13 are consistent with the results of western blotting (Figures 3C,D). In summary, these results indicate that Sin has the effect of protecting chondrocytes from TBHP-induced ECM degradation.

### Effect of Sin on Autophagy in Chondrocytes

**FIGURE 4:**
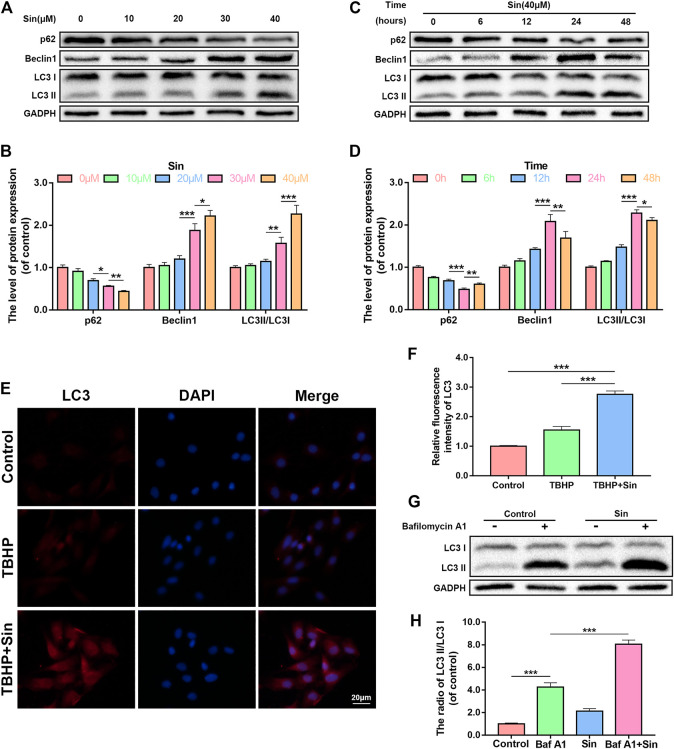
Sin activates autophagy in chondrocytes. **(A, B)** The results of dose-dependent western blotting show that the levels of p62, Beclin1, and LC3II/LC3I ratio in chondrocytes treated with different concentrations of Sin (0, 10, 20, 30, and 40 μM) for 24 h. **(C, D)** The results of time-dependent western blotting show that the levels of p62, Beclin1, and LC3II/LC3I ratio in chondrocytes treated with 40 μM Sin for different times (0, 6, 12, 24,and 48 h). **(E, F)** The results of immunofluorescence show the intensity of LC3 in the chondrocytes treated with or without 40 μM Sin for 24 h and 30 μM TBHP for 24 h (Scale bar: 20 μm). **(G, H)** The western blotting results show that the levels of LC3II/LC3I ratio in chondrocytes that were not treated, or treated with 100 nM bafilomycin A1 for 1 h, or treated with 40 μM Sin for 24 h, or treated with bafilomycin A1 and Sin. The data are presented as the means ± SD (*n* = 3); *p < 0.05, **p < 0.01, and ***p < 0.001.

Autophagy is an intracellular degradation mechanism, which transports cytoplasmic components to the lysosome for degradation, and plays an important role in the progression of OA (Mizushima, 2007; Li et al., 2016). We analyzed whether Sin activates autophagy in chondrocytes by detecting autophagy markers, such as p62, Beclin1, and LC3II/LC3I ratio, using a dose- and time-dependent method. Dose-dependent western blotting showed that Sin treatment increases the expression of Beclin1 and the ratio of LC3II/LC3I, while p62 expression decreased (Figures 4A,B). And the immunofluorescence results of labeled LC3 showed the same result (Figures 4E,F). And time-dependent western blotting showed that Sin treatment increased the expression of Beclin1 and the ratio of LC3II/LC3I, and reached a peak at 24 h. On the contrary, the expression of p62 after Sin treatment gradually decreased (Figures 4C,D).

In addition, autophagy flux is also a reliable indicator for detecting autophagy (Zheng et al., 2018). By treating chondrocytes with Sin and the lysosomal inhibitor bafilomycin A1, we analyzed whether Sin also enhances autophagy flux. As shown in Figures 4G,H, the autophagy flux of chondrocytes increased after Sin treatment.

### Sin Activates the AMPK/mTOR Pathway in Chondrocytes

**FIGURE 5:**
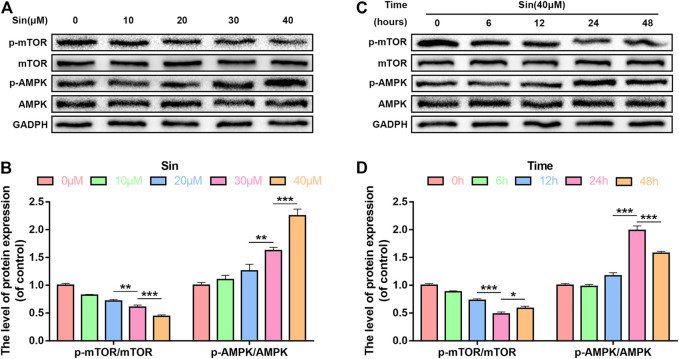
Sin activates the AMPK/mTOR pathway in chondrocytes. **(A, B)** The results of dose-dependent western blotting show that the levels of p-mTOR and p-AMPK in chondrocytes treated with different concentrations of Sin (0, 10, 20, 30, and 40 μM) for 24 h. **(C, D)** The results of time-dependent western blotting show that the levels of p-mTOR and p-AMPK in chondrocytes treated with 40 μM Sin for different times (0, 6, 12, 24,and 48 h). The data are presented as the means ± SD (*n* = 3); *p < 0.05, **p < 0.01, and ***p < 0.001.

AMP-activated protein kinase (AMPK) is a key regulator of autophagy by inhibiting mammalian target of rapamycin C1 (mTORC1) (Lahiri et al., 2019). In order to study whether the mechanism of Sin’s activation of autophagy is related to the AMPK/mTOR signaling pathway, we used western blot to detect the expression levels of p-AMPK and p-mTOR. The dose-dependent western blotting showed that after Sin treatment, the expression of p-AMPK in chondrocytes is significantly increased, but the expression of total AMPK is not affected, and the expression of p-mTOR is decreased (Figures 5A,B). Additionally, time-dependent western blotting showed that Sin treatment increases the expression of p-AMPK and decreases the expression of p-mTOR (Figures 5C,D). These results indicate that Sin activates the AMPK/mTOR pathway, and the autophagy activated by Sin may be related to the AMPK/mTOR pathway.

### Inhibition of Autophagy Attenuates Sin-Induced Anti-apoptosis and Anti-ECM Degradation

**FIGURE 6:**
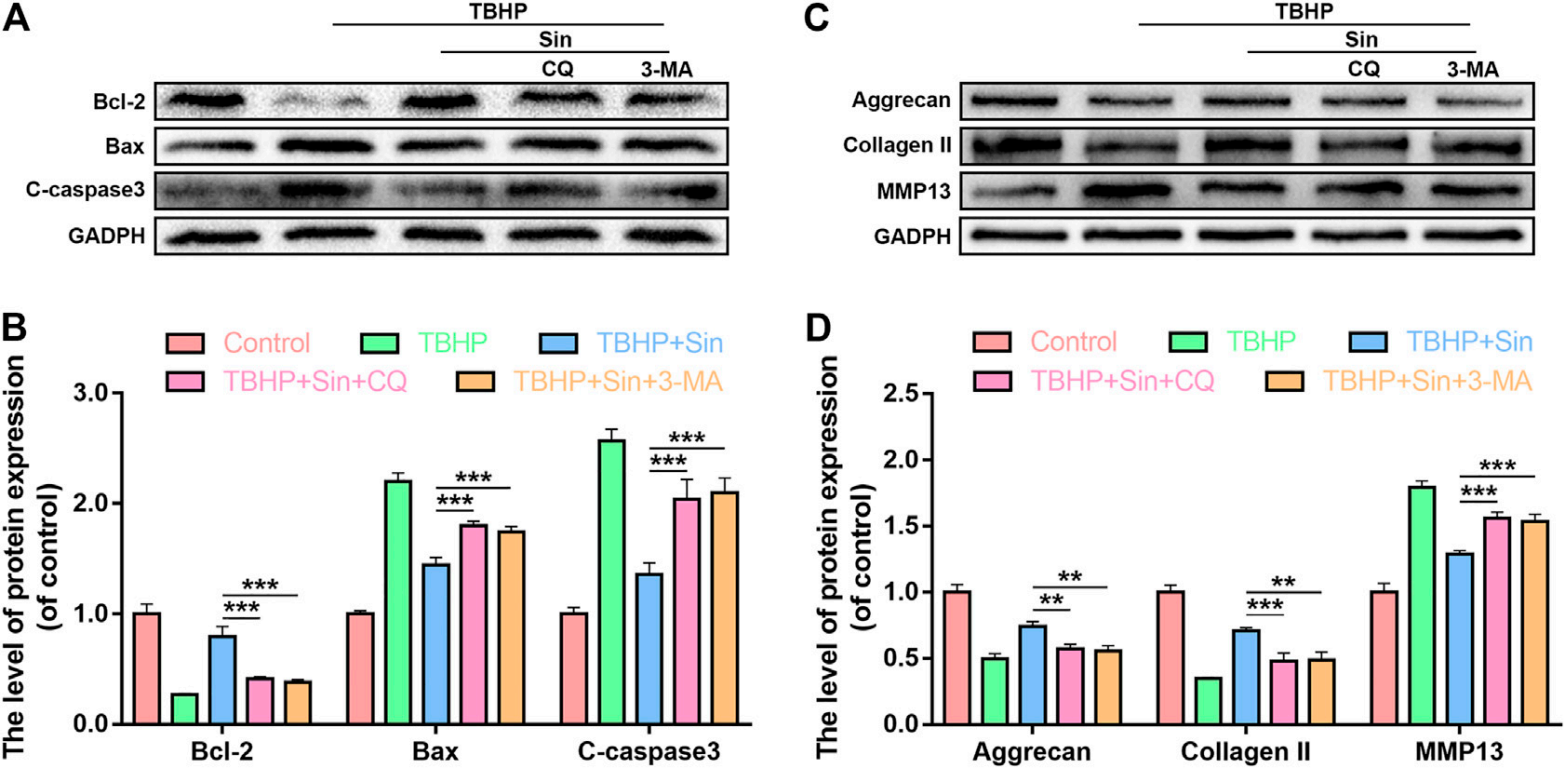
Inhibition of autophagy offsets the beneficial effect of Sin. Chondrocytes were untreated, or treated with TBHP (30 μM for 24 h) alone, or treated with Sin (40 μM for 24 h) and TBHP, or treated with TBHP and Sin combined with CQ (50 μM for 2 h) or 3-MA (10 mM for 2 h). **(A, B)** The results of western blotting show that the levels of Bcl-2, Bax and cleaved caspase 3 in chondrocytes treated above. **(C, D)** The results of western blotting show that the levels of Aggrecan, collagen II and MMP13 in chondrocytes treated above. The data are presented as the means ± SD (n = 3); *p < 0.05, **p < 0.01, and ***p < 0.001.

To further prove that Sin protects chondrocytes from apoptosis and ECM degradation by activating autophagy, we used two autophagy inhibitors Chloroquine (CQ) and 3-Methyladenine (3-MA) to block autophagy. The results of western blotting showed that CQ or 3-MA significantly increased the expression of cleaved caspase 3 and Bax, and decreased the expression of Bcl-2 in Sin-treated chondrocytes (Figures 6A,B). As shown in Figures 6C,D, in Sin-treated chondrocytes, CQ or 3-MA also significantly increased the expression of MMP13 and decreased the expression of Aggrecan and Collagen II. The above results indicate that Sin inhibits chondrocyte apoptosis and ECM degradation by enhancing chondrocytes autophagy.

### Sin Ameliorates *in vivo* OA Progression in DMM Model Mice

**FIGURE 7:**
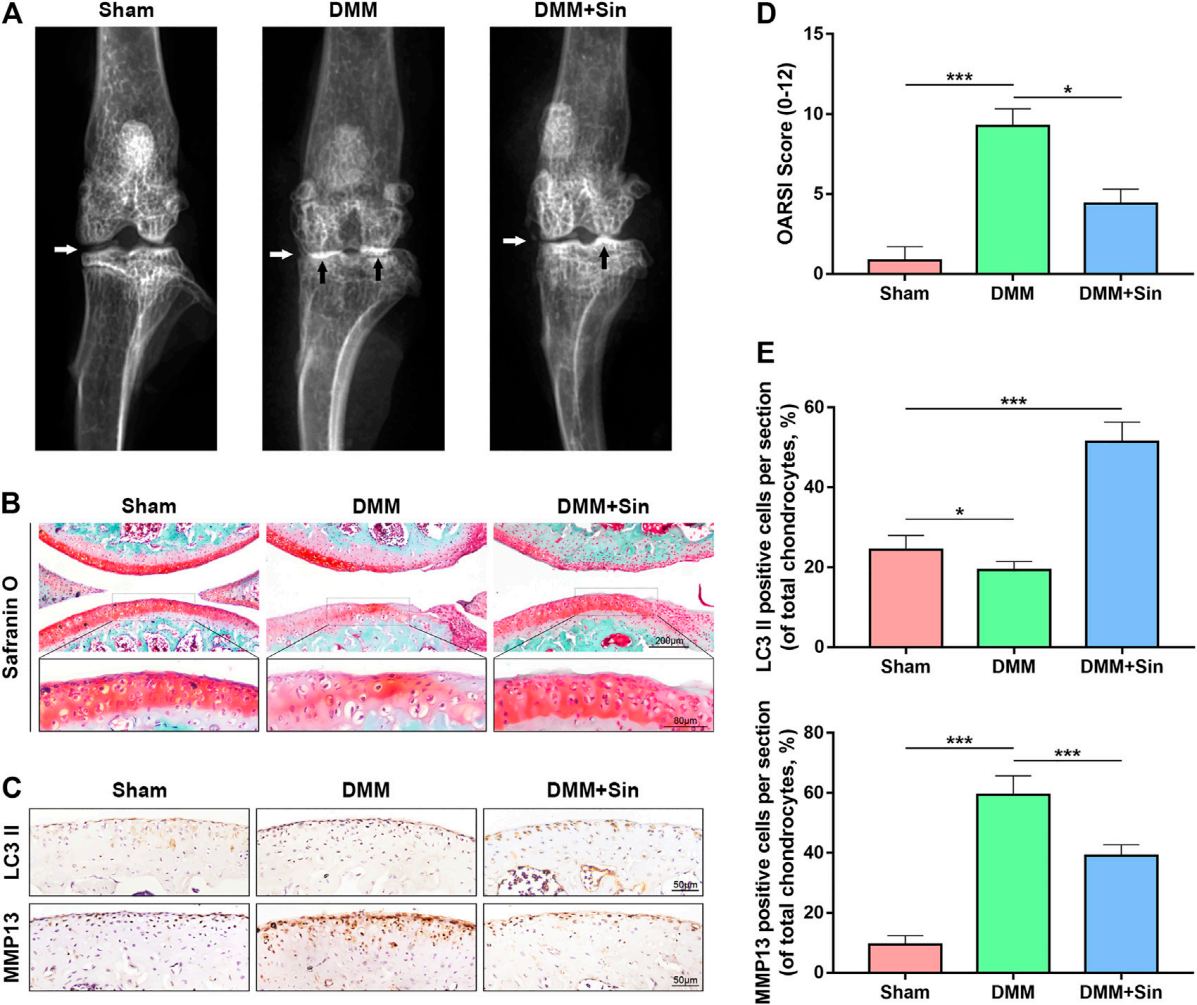
Sin protects against DMM-induced OA pathogenesis. **(A)** Representative X-ray images of the knee joints of mice in different experimental groups. White arrows indicate narrowing of the joint space, and black arrows indicate calcification of the cartilage surface. **(B)** Representative images of cartilage S-O staining in the three groups at 8 weeks postoperatively. **(C)** Immunohistochemical staining assay of LC3 II and MMP13 in the mouse cartilage (Scale bar: 50 μm). **(D)** OARIS scores of articular cartilage in three groups. **(E)** Quantitative analysis of LC3 II and MMP13 positive expression in sections. The data are presented as the means ± SD (*n* = 10); *p < 0.05, **p < 0.01, and ***p < 0.001.

In order to investigate the therapeutic effect of Sin on OA *in vivo*, we administered Sin to DMM mice by gavage. We established the OA model in mice by DMM, and for the following 8 weeks, the DMM mice were given Sin (Sin group) or saline (DMM group) by gavage once a day, and the joints were analyzed by X-ray, safranin O staining and immunofluorescence. The results of X-ray showed that the joint space was severely narrowed and cartilage was obviously ossified in DMM mice, while the joint space narrowing and cartilage solidification were improved after Sin treatment (Figure 7A). Eight weeks after surgery, safranin O staining showed that the joint surface was rough and chondrocytes were reduced in the DMM group, but Sin treatment partially rescued these conditions (Figure 7B). Consistent with the staining results, Sin treatment reduced the Osteoarthritis Research Society International (OARSI) score (Figure 7D).

The results of immunohistochemistry showed that the Sin treatment group could effectively reduce the expression of MMP13 and increase the expression of LC3. These results indicate that Sin improves the OA situation and reduces the degradation of ECM by enhancing the autophagy of chondrocytes in the OA mice (Figures 7C,E).

## Discussion

Osteoarthritis (OA) is a common degenerative chronic disease that lacks effective treatment methods (Nelson, 2018). In this study, we investigated the role of Sinensetin (Sin) in alleviating the progression of OA and its underlying mechanism. We found that Sin not only inhibited apoptosis and extracellular matrix (ECM) degradation in TBHP-induced chondrocytes, but also promoted the autophagy function of chondrocytes. In addition, we further proved that Sin enhances autophagy through AMPK/mTOR signaling pathway, thereby ameliorating OA.

Previous studies have shown that Sin is a plant extract with good antioxidant (Yao et al., 2009), anti-inflammatory (Chae et al., 2017) and antimicrobial properties (Vikram et al., 2013). Pathological factors such as inflammation and oxidative stress are involved in the occurrence and development of OA (Goutas et al., 2018; Wang and He, 2018), and reactive oxygen species (ROS) play an important role in the pathology of OA (Lepetsos and Papavassiliou, 2016). Therefore, we speculate that Sin may weaken ROS-induced apoptosis and ECM degradation in chondrocytes, and ameliorate the progression of OA. First, we found that Sin at a concentration of less than 40 M has no significant cytotoxicity to chondrocytes. Subsequently, we demonstrated that Sin protects TBHP (as ROS donor)-induced chondrocytes from apoptosis and ECM degradation, and activates chondrocyte autophagy.

Apoptosis of chondrocytes causes OA cartilage destruction (Charlier et al., 2016), so inhibiting apoptosis of chondrocytes protects against OA pathogenesis (Son et al., 2019). We thus investigated whether Sin has a protective effect on chondrocyte apoptosis. Our experimental results show that Sin reduces the apoptosis of chondrocytes treated by TBHP, indicating Sin’s therapeutic potential for OA.

The up-regulation of matrix degrading enzymes and/or the degradation of cartilage extracellular matrix (ECM) is an important cause of cartilage destruction (Troeberg and Nagase, 2012). Our research proves that Sin reduces the level of matrix metalloproteinase 13 (MMP13) protein and increases the level of collagen II and aggrecan in TBHP-treated chondrocytes, indicating that Sin can maintain the homeostasis of ECM.

Autophagy is the degradation process of cells, which helps maintain cell homeostasis and improve cell survival and function (Mizushima, 2007). Autophagy has been proved to be a potential therapeutic target for OA, because it helps to alleviate the apoptosis and senescence of chondrocytes (Appleton, 2018). Previous studies have shown that Sin is an autophagy inducer used to treat colitis and hepatocellular carcinoma (Xiongjian et al., 2019; Kim et al., 2020). In our study, Sin also significantly improved the autophagy function of chondrocytes, and its protective effect on TBHP-treated chondrocytes was abrogated when autophagy was inhibited, indicating that Sin plays a protective role by enhancing autophagy.

A variety of upstream signaling pathways are involved in the activation of autophagy. Among them, AMP activated protein kinase (AMPK), as a metabolic sensor, plays an important role in regulating autophagy (Mihaylova and Shaw, 2011). In addition, mTOR is a negative regulator of autophagy, and its activity is involved in AMPK-mediated autophagy (Klionsky et al., 2016). Our results showed that Sin activates AMPK activity and reduces mTOR activity in chondrocytes in a time- and dose-dependent manner, indicating that Sin may activate autophagy through AMPK/mTOR signaling pathway.

**FIGURE 8:**
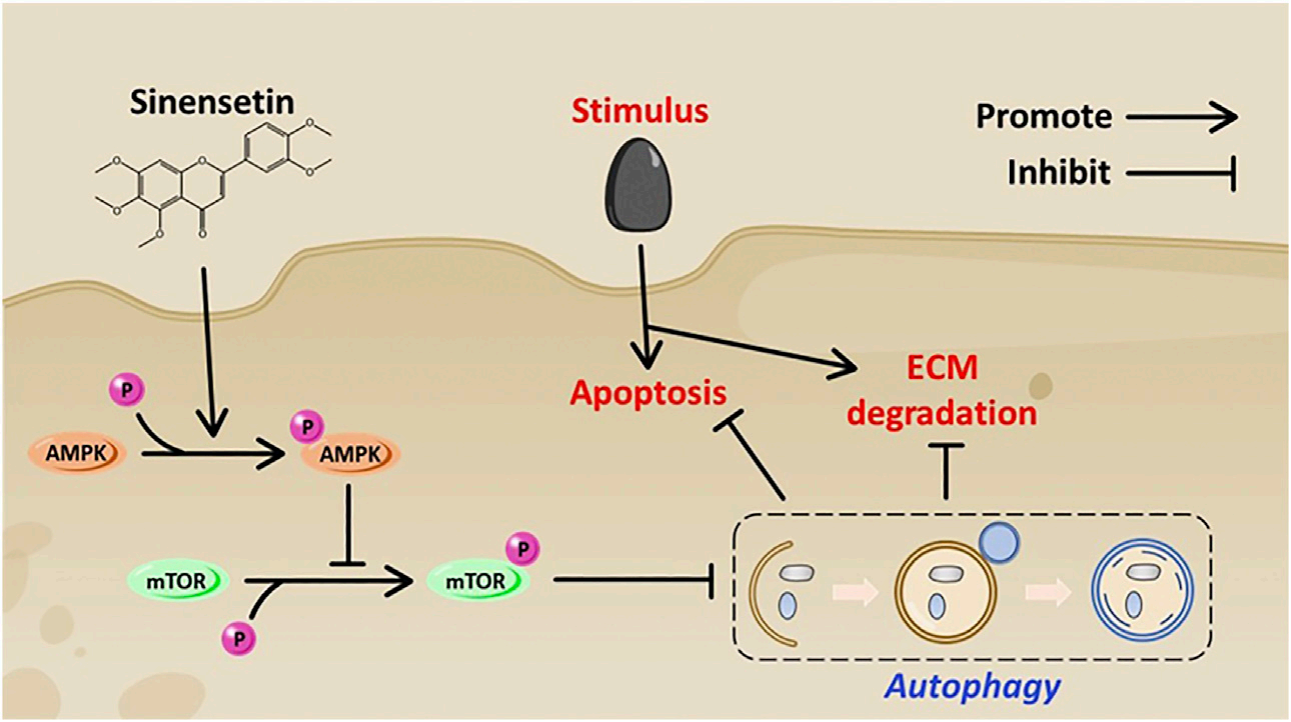
Schematic diagram shows the potential protective effect of Sin on OA.

Our research has several limitations. We cannot establish a direct interaction between Sin and AMPK, and we should verify the *in vivo* effects of Sin in joint-specific knockdown AMPK mice. Another shortcoming of our study is that there are a few indicators to evaluate the protective effect of Sin on OA *in vivo*. Further *in vivo* characterization and functional studies are needed to prove the therapeutic effect of Sin on OA. In conclusion, our research shows that Sin activates autophagy *via* AMPK/mTOR signaling pathway, thereby effectively inhibiting the apoptosis and ECM degradation of TBHP-treated chondrocytes and the pathogenesis of OA in DMM model mice (Figure 8).

## Data Availability

The original contributions presented in the study are included in the article/Supplementary Material, further inquiries can be directed to the corresponding author.

## References

[B1] Appleton C. T. (2018). Osteoarthritis Year in Review 2017: Biology. Osteoarthritis and Cartilage 26, 296–303. 10.1016/j.joca.2017.10.008 29061493

[B2] Caramés B. Olmer M. Kiosses W. B. Lotz M. K. (2015). The Relationship of Autophagy Defects to Cartilage Damage during Joint Aging in a Mouse Model. Arthritis Rheumatol. 67, 1568–1576. 10.1002/art.39073 25708836 PMC4446178

[B3] Chae H.-S. Kim Y.-M. Chin Y.-W. (2017). Sinensetin Inhibits Interleukin-6 in Human Mast Cell - 1 via Signal Transducers and Activators of the Transcription 3 (STAT3) and Nuclear Factor Kappa B (NF-Κb) Pathways. Nat. Prod. Sci. 23, 1. 10.20307/nps.2017.23.1.1

[B4] Charlier E. Relic B. Deroyer C. Malaise O. Neuville S. Collée J. (2016). Insights on Molecular Mechanisms of Chondrocytes Death in Osteoarthritis. Ijms 17, 2146. 10.3390/ijms17122146 PMC5187946 27999417

[B5] Glasson S. S. Blanchet T. J. Morris E. A. (2007). The Surgical Destabilization of the Medial Meniscus (DMM) Model of Osteoarthritis in the 129/SvEv Mouse. Osteoarthritis and Cartilage 15, 1061–1069. 10.1016/j.joca.2007.03.006 17470400

[B6] Glasson S. S. Chambers M. G. Van Den Berg W. B. Little C. B. (2010). The OARSI Histopathology Initiative - Recommendations for Histological Assessments of Osteoarthritis in the Mouse. Osteoarthritis and Cartilage 18, S17–S23. 10.1016/j.joca.2010.05.025 20864019

[B7] Goutas A. Syrrou C. Papathanasiou I. Tsezou A. Trachana V. (2018). The Autophagic Response to Oxidative Stress in Osteoarthritic Chondrocytes Is Deregulated. Free Radic. Biol. Med. 126, 122–132. 10.1016/j.freeradbiomed.2018.08.003 30096432

[B8] Han Jie L. Jantan I. Yusoff S. D. Jalil J. Husain K. (2021). Sinensetin: An Insight on its Pharmacological Activities, Mechanisms of Action and Toxicity. Front. Pharmacol. 11, 1–16. 10.3389/fphar.2020.553404 PMC7898666 33628166

[B9] Kim S. M. Ha S. E. Lee H. J. Rampogu S. Vetrivel P. Kim H. H. (2020). Sinensetin Induces Autophagic Cell Death through P53-Related AMPK/mTOR Signaling in Hepatocellular Carcinoma HepG2 Cells. Nutrients 12, 2462. 10.3390/nu12082462 PMC7468969 32824273

[B10] Klionsky D. J. Abdelmohsen K. Abe A. Abedin M. J. Abeliovich H. Arozena A. A. (2016). Guidelines for the Use and Interpretation of Assays for Monitoring Autophagy. Autophagy 12, 1–222. 10.1080/15548627.2015.110035610.1080/15548627.2016.1139264 3rd edition 26799652 PMC4835977

[B11] Kronenberg H. M. (2003). Developmental Regulation of the Growth Plate. Nature 423, 332–336. 10.1038/nature01657 12748651

[B12] Lahiri V. Hawkins W. D. Klionsky D. J. (2019). Watch what You (Self-) Eat: Autophagic Mechanisms that Modulate Metabolism. Cel Metab. 29, 803–826. 10.1016/j.cmet.2019.03.003 PMC6450419 30943392

[B13] Lepetsos P. Papavassiliou A. G. (2016). ROS/oxidative Stress Signaling in Osteoarthritis. Biochim. Biophys. Acta (Bba) - Mol. Basis Dis. 1862, 576–591. 10.1016/j.bbadis.2016.01.003 26769361

[B14] Li J. Jie X. Liang X. Chen Z. Xie P. Pan X. (2020). Sinensetin Suppresses Influenza a Virus-Triggered Inflammation through Inhibition of NF-Κb and MAPKs Signalings. BMC Complement. Med. Ther. 20, 135. 10.1186/s12906-020-02918-3 32370749 PMC7200050

[B15] Li Y.-S. Zhang F.-J. Zeng C. Luo W. Xiao W.-F. Gao S.-G. (2016). Autophagy in Osteoarthritis. Jt. Bone Spine 83, 143–148. 10.1016/j.jbspin.2015.06.009 26453105

[B16] Mihaylova M. M. Shaw R. J. (2011). The AMPK Signalling Pathway Coordinates Cell Growth, Autophagy and Metabolism. Nat. Cel Biol. 13, 1016–1023. 10.1038/ncb2329 PMC3249400 21892142

[B17] Mizushima N. (2007). Autophagy: Process and Function. Genes Development 21, 2861–2873. 10.1101/gad.1599207 18006683

[B18] Nelson A. E. (2018). Osteoarthritis Year in Review 2017: Clinical. Osteoarthritis and Cartilage 26, 319–325. 10.1016/j.joca.2017.11.014 29229563 PMC5835411

[B19] Safiri S. Kolahi A.-A. Smith E. Hill C. Bettampadi D. Mansournia M. A. (2020). Global, Regional and National burden of Osteoarthritis 1990-2017: a Systematic Analysis of the Global Burden of Disease Study 2017. Ann. Rheum. Dis. 79, 819–828. 10.1136/annrheumdis-2019-216515 32398285

[B20] Son Y.-O. Kim H.-E. Choi W.-S. Chun C.-H. Chun J.-S. (2019). RNA-binding Protein ZFP36L1 Regulates Osteoarthritis by Modulating Members of the Heat Shock Protein 70 Family. Nat. Commun. 10. 10.1038/s41467-018-08035-7 PMC6325149 30622281

[B21] Tan K.-T. Lin M.-X. Lin S.-C. Tung Y.-T. Lin S.-H. Lin C.-C. (2019). Sinensetin Induces Apoptosis and Autophagy in the Treatment of Human T-Cell Lymphoma. Anticancer. Drugs 30, 485–494. 10.1097/CAD.0000000000000756 30702500

[B22] Troeberg L. Nagase H. (2012). Proteases Involved in Cartilage Matrix Degradation in Osteoarthritis. Biochim. Biophys. Acta (Bba) - Proteins Proteomics 1824, 133–145. 10.1016/j.bbapap.2011.06.020 21777704 PMC3219800

[B23] van den Bosch M. H. J. (2021). Osteoarthritis Year in Review 2020: Biology. Osteoarthritis and Cartilage 29, 143–150. 10.1016/j.joca.2020.10.006 33242602

[B24] Vikram A. Jayaprakasha G. K. Uckoo R. M. Patil B. S. (2013). Inhibition of *Escherichia coli* O157:H7 Motility and Biofilm by β-Sitosterol Glucoside. Biochim. Biophys. Acta (Bba) - Gen. Subjects 1830, 5219–5228. 10.1016/j.bbagen.2013.07.022 23891936

[B25] Vina E. R. Kwoh C. K. (2018). Epidemiology of Osteoarthritis: Literature Update. Curr. Opin. Rheumatol. 30, 160–167. 10.1097/BOR.0000000000000479 29227353 PMC5832048

[B26] Wang T. He C. (2018). Pro-inflammatory Cytokines: The Link between Obesity and Osteoarthritis. Cytokine Growth Factor. Rev. 44, 38–50. 10.1016/j.cytogfr.2018.10.002 30340925

[B27] Xiong Y.-j. Deng Z.-b. Liu J.-n. Qiu J.-j. Guo L. Feng P.-p. (2019). Enhancement of Epithelial Cell Autophagy Induced by Sinensetin Alleviates Epithelial Barrier Dysfunction in Colitis. Pharmacol. Res. 148, 104461. 10.1016/j.phrs.2019.104461 31542404

[B28] Yao X. Xu X. Fan G. Qiao Y. Cao S. Pan S. (2009). Determination of Synergistic Effects of Polymethoxylated Flavone Extracts of Jinchen orange Peels (Citrus Sinensis Osberk) with Amino Acids and Organic Acids Using Chemiluminescence. Eur. Food Res. Technol. 229, 743–750. 10.1007/s00217-009-1100-6

[B29] Zhang Y. Vasheghani F. Li Y.-H. Blati M. Simeone K. Fahmi H. (2015). Cartilage-specific Deletion of mTOR Upregulates Autophagy and Protects Mice from Osteoarthritis. Ann. Rheum. Dis. 74, 1432–1440. 10.1136/annrheumdis-2013-204599 24651621

[B30] Zheng G. Zhan Y. Li X. Pan Z. Zheng F. Zhang Z. (2018). TFEB, a Potential Therapeutic Target for Osteoarthritis via Autophagy Regulation. Cell Death Dis. 9. 10.1038/s41419-018-0909-y PMC6113230 30154423

