# Supplementary material for: Sinensetin Reduces Osteoarthritis Pathology in the Tert-Butyl Hydroperoxide-Treated Chondrocytes and the Destabilization of the Medial Meniscus Model Mice via the AMPK/mTOR Signaling Pathway
Source: Front Pharmacol. 2021 Jul 16;12:713491. doi: 10.3389/fphar.2021.713491 (PMC8322586; doi:10.3389/fphar.2021.713491)
Supplement: Supplementary file 1 [file DataSheet1.PDF]

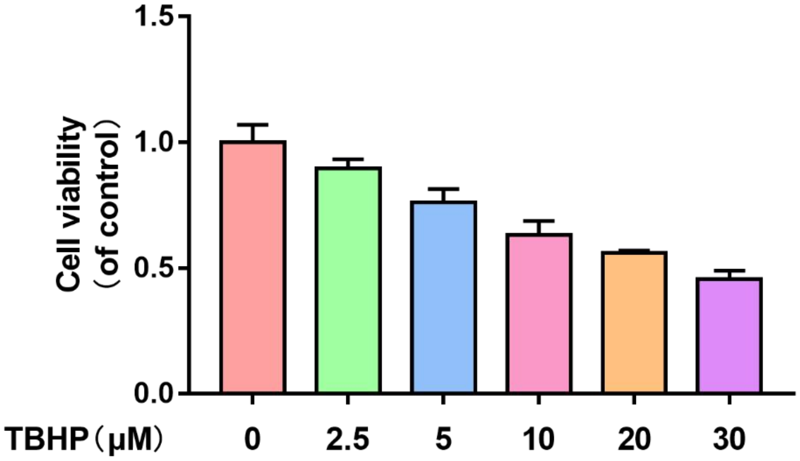

Supplementary Figure 1. The results of CCK8 assay show the viability of chondrocytes treated with different concentrations of TBHP for 24h.
